# Antibacterial activity of novel linear polyamines against *Staphylococcus aureus*

**DOI:** 10.3389/fmicb.2022.948343

**Published:** 2022-08-22

**Authors:** Edward J. A. Douglas, Abdulaziz H. Alkhzem, Toska Wonfor, Shuxian Li, Timothy J. Woodman, Ian S. Blagbrough, Maisem Laabei

**Affiliations:** ^1^Department of Biology and Biochemistry, University of Bath, Bath, United Kingdom; ^2^Department of Pharmacy and Pharmacology, University of Bath, Bath, United Kingdom

**Keywords:** polyamines, *Staphylococcus aureus*, antibacterial activity, antibiotic synergy, antimicrobial resistance (AMR)

## Abstract

New therapeutic options are urgently required for the treatment of *Staphylococcus aureus* infections. Accordingly, we sought to exploit the vulnerability of *S. aureus* to naturally occurring polyamines. We have developed and tested the anti-staphylococcal activity of three novel linear polyamines based on spermine and norspermine. Using a panel of genetically distinct and clinically relevant multidrug resistant *S. aureus* isolates, including the polyamine resistant USA300 strain LAC, compound AHA-1394 showed a greater than 128-fold increase in inhibition against specific *S. aureus* strains compared to the most active natural polyamine. Furthermore, we show that AHA-1394 has superior biofilm prevention and biofilm dispersal properties compared to natural polyamines while maintaining minimal toxicity toward human HepG2 cells. We examined the potential of *S. aureus* to gain resistance to AHA-1394 following *in vitro* serial passage. Whole genome sequencing of two stable resistant mutants identified a gain of function mutation (S337L) in the phosphatidylglycerol lysyltransferase *mprF* gene. Inactivation of mutant *mprF* confirmed the importance of this allele to AHA-1394 resistance. Importantly, AHA-1394 resistant mutants showed a marked decrease in relative fitness and increased generation time. Intriguingly, *mprF*::S337L contributed to altered surface charge only in the USA300 background whereas increased cell wall thickness was observed in both USA300 and SH1000. Lastly, we show that AHA-1394 displays a particular proclivity for antibiotic potentiation, restoring sensitivity of MRSA and VRSA isolates to daptomycin, oxacillin and vancomycin. Together this study shows that polyamine derivatives are impressive drug candidates that warrant further investigation.

## Introduction

*Staphylococcus aureus* has become notorious as a multidrug resistant pathogen. Globally, an estimated 100,000 deaths are attributed to, and almost 1 million deaths associated with resistant *S. aureus* infections, second only to *Escherichia coli* in AMR burden (Murray et al., 2022).

Vancomycin (glycopeptide) and daptomycin (lipopeptide) remain the treatments of choice for invasive MRSA infections (Catherine Liu et al., 2011). Vancomycin failure is increasingly being reported in MRSA infections and is associated with a slow increase in vancomycin minimal inhibitory concentrations (MIC), coined “MIC creeping” (Canty et al., 2021). Similarly, daptomycin resistant strains of MRSA have been reported, and have been attributed to single nucleotide polymorphisms (SNPs) housed in cell envelope and two-component system genes (Miller et al., 2016). As such, the current treatment options for MRSA, particularly invasive infections, are limited.

Polyamines are small polycationic molecules that are involved in numerous cellular functions both in eukaryotes and bacteria (Hamana and Matsuzaki, 1992). The uniform distribution of positive charges across a hydrophobic backbone are thought to be essential in how these compounds exert their diverse array of functions (Cohen, 1998). Polyamines interact with highly anionic molecules such as nucleic acids, phospholipids and certain proteins leading to pleiotropic effects in gene expression, membrane stabilization, enzymatic activity, and protein translation (Tabor and Tabor, 1984; Schuber, 1989; Zheliaskova et al., 2000; Sakamoto et al., 2020). Until recently it was believed that polyamines were produced and required by all forms of life. However, certain bacterial species lack *de novo* polyamine biosynthesis machinery and rely on polyamine import from their host. In these cases, supplementation of growth medium with polyamines such as spermine and spermidine enhance bacterial growth (Joshi et al., 2011). *S. aureus* is a peculiar example as it lacks polyamine biosynthetic genes and displays hypersensitivity to exogenously added polyamines, where treatment with spermine and spermidine was shown to be bactericidal (Joshi et al., 2011; Thurlow et al., 2013).

**FIGURE 1:**
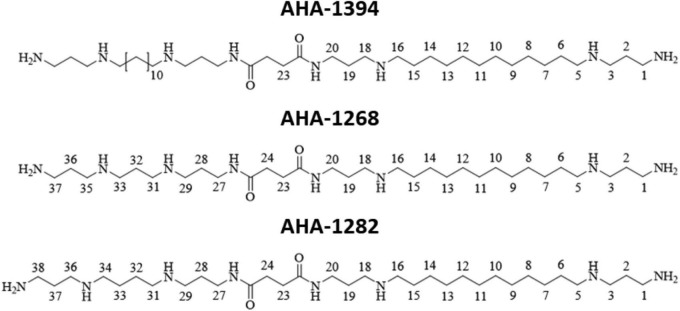
Structures of novel linear polyamines with improved anti-staphylococcal activity used in this study.

The development of new antimicrobials and anti-infectives represents an important step in combatting antimicrobial resistance as outlined by the UN Interagency Coordination Group on AMR (The Ad hoc Interagency Coordination Group (IACG) on Antimicrobial Resistance, 2019). Accordingly, in this study we sought to exploit the existing sensitivity of *S. aureus* toward polyamines, synthesizing novel linear polyamine compounds (Figure 1) which had superior anti-staphylococcal activity compared to natural compounds. One of these polyamine derivatives, designated AHA-1394, displayed potent bactericidal activity against a range of *S. aureus* clinical isolates, including hyper virulent community-associated MRSA, vancomycin intermediate *S. aureus* (VISA) and vancomycin resistant *S. aureus* (VRSA) strains, whilst displaying mild toxigenic effects to host cells comparably to clinical used antibiotics. Gene inactivation of *mprF* resulted in enhanced susceptibility whereas *mprF* gain of function mutations, specifically S337L, conferred resistance. Finally, we report significant synergism of AHA-1394 with oxacillin, vancomycin and daptomycin using a panel of multidrug resistant *S. aureus* isolates, highlighting the potential of AHA-1394 to resensitize *S. aureus* to clinically relevant antibiotics.

## Materials and methods

### Bacterial strains and culture conditions

*S. aureus* strains used in this study as listed in Table 1. *S. aureus* isolates were streaked onto tryptic soy agar (TSA) and incubated for 18 h at 37°C, and single colonies transferred into 3 ml of growth medium in 15 ml polystyrene test tubes. Erythromycin (5 μg/ml) was added to the growth medium for transposon mutants obtained from the Nebraska Transposon Mutant Library (NTML) (Fey et al., 2013) DNA from JE2 *mprF::Tn* was transduced into SH1000-B and SH1000-128 with Φ11 and colonies containing the inserted transposon were screened on TSA containing erythromycin (10 μg/ml).

**TABLE 1:** List of strains used in this study.

Strain	Description	References
SH1000	MSSA, laboratory strain, 8325-4 with a repaired *rsbU* gene; SigB positive (CC8)	Horsburgh et al., 2002
Newman	MSSA, laboratory strain isolated from human infection (CC8), lacks antibiotic resistant determinants	Hawiger et al., 1970
Newman Δ*menD*	A *menD* deficient strain in the Newman background. The *menD* gene contains a K253STOP substitution	Altwiley et al., 2021
MRSA252	HA-MRSA type II SCC*mec* (CC30) resistant to penicillin, ciprofloxacin, erythromycin, and methicillin and sensitive to fusidic acid, rifampicin, tetracycline, trimethoprim, gentamicin, and amikacin	Holden et al., 2004
TW20	HA-MRSA type III SCC*mec* (CC239), resistant to penicillin, methicillin, erythromycin, ciprofloxacin, gentamicin, neomycin, trimethoprim, and tetracycline	Holden et al., 2010
EMRSA15	HA/CA-MRSA type IV SCC*mec* (CC22), resistant to penicillin, methicillin, erythromycin, ciprofloxacin, tetracycline, trimethoprim, gentamicin, fusidic acid, mupirocin	Richardson and Reith, 1993
LAC	CA-MRSA USA300 type IV SCC*mec* (CC8), resistant to penicillin, methicillin, erythromycin, ciprofloxacin, clindamycin, streptogramin B, tetracycline, mupirocin	Diep et al., 2006
MW2	CA-MRSA USA400 type IV SCC*mec* (CC1), resistant to penicillin, methicillin	Baba et al., 2002
Mu3	Clinical isolate hetero-VISA type II SCC*mec* (CC5), resistant to methicillin	Hiramatsu et al., 1997
Mu50	MRSA clinical isolate (Japan 1997)—Vancomycin resistant type II SCC*mec* (CC5)	Hiramatsu et al., 1997
JE2	CA -MRSA USA300 type IV SCC*mec*; lacking plasmids p01 and p03; wild-type strain of the NTML (CC8)	Fey et al., 2013
JE2 *speG::Tn*	*speG bursa aurealis* transposon mutant in the JE2 background	Fey et al., 2013
JE2 *mprF::Tn*	*mprF bursa aurealis* transposon mutant in the JE2 background	Fey et al., 2013
SH1000-B	SH1000 passaged in Mueller Hinton broth	This study
SH1000-B *mprF::Tn*	*mprF bursa aurealis* transposon mutant in the SH1000-B background	This study
SH1000-128	SH1000 passaged in Mueller Hinton broth + AHA-1394 to a final concentration of 128 μg/ml	This study
SH1000-128 *mprF::Tn*	*mprF bursa aurealis* transposon mutant in the SH1000-128 background	This study
LAC-B	LAC passaged in Mueller Hinton broth	This study
LAC-128	LAC passaged in Mueller Hinton broth + AHA-1394 to a final concentration of 128 μg/ml	This study

### Synthesis of polyamine compounds

A two-step procedure was used to form long linear tetraamines (Karigiannis and Papaioannou, 2000) from commercially available 1,12-diaminododecane by reaction with two equivalents of acrylonitrile to undergo two 1,4-Michael addition reactions to obtain the dinitrile in 87% yield (Figure 1). Both the nitrile functional groups were successfully reduced to primary amines using catalytic Raney nickel and sodium hydroxide (co-catalyst) under a hydrogen pressure of 1 bar (Bergeron and Garlich, 1984; Klenke and Gilbert, 2001) to obtain the desired tetraamine in 75 % yield. Trifluoroacetyl is an ideal amine protecting group in the gram-scale protection of polyamines in preparing unsymmetrical polyamine amides (Geall and Blagbrough, 2000). The key intermediate carboxylic acids were then synthesized by the addition of one equivalent of succinic anhydride to a solution of the triBoc-protected long linear polyamines in anhydrous pyridine. All spectral data confirmed that the reactions successfully occurred. The designed target polyamine amides hetero AHA-1268 and AHA-1282, and homo AHA-1394 dimers of these linear polyamine amides incorporating one or two units of 1,12-diaminododecane as 3.12.3 methylene moieties were prepared on a 100–300 mg scale in good yield, and the final poly-TFA salt products were lyophilized and confirmed by mass spectrometry.

AHA-1268 HRMS: Found 585.5538 (*m/z*), C_31_H_69_N_8_O_2_ requires 585.5465 (*m/z*) [M+H]^+^.
AHA-1282 HRMS: Found 599.5690 (*m/z*), C_32_H_71_N_8_O_2_ requires 599.5630 (*m/z*) [M+H]^+^.
AHA-1394 HRMS: Found 733.6757 (*m/z*), C_40_H_86_NaN_8_O_2_ requires 733.6874 (*m/z*) [M+Na]^+^.

### Determination of polyamine minimum inhibitory concentration

Minimum inhibitory concentration (MIC) of polyamine compounds against *S. aureus* strains were determined by the microdilution method using Mueller Hinton broth (MHB) (CLSI, 2018). Polyamine compounds were reconstituted in sterile deionized water and diluted in MHB, to a final concentration ranging between 256 and 0.5 μg/ml. The MIC was determined as the lowest concentration that completely inhibited bacterial growth. Experiments were performed using two technical repeats with three biological replicates.

### Biofilm prevention and dispersion assays

*S. aureus* strain SH1000 was used in all biofilm studies. For biofilm prevention, 18 h bacterial cultures were diluted 1:200 in Tryptic soy broth containing 0.5% glucose (TSB-G) and added to polyamines in a 1:1 ratio to a final concentration ranging between 256 and 2 μg/ml in 96-well flat-bottomed microtiter plate, incubated for 24 h at 37°C under static conditions. For biofilm dispersion assays, 18 h bacterial cultures were diluted 1:400 in TSB-G in 96 well plates and incubated at 37°C for 6 h or 24 h, following which non-adhered bacteria were carefully aspirated and biofilms washed three times in sterile PBS. Following washing, 200 μl of individual polyamines were diluted in TSB-G and added to biofilms to a final concentration ranging between 4,096 and 8 μg/ml and incubated for a further 24 h 37°C under static conditions. Biofilm formation was determined using the crystal violet assay as previously described (Lacoma et al., 2019). Experiments were performed with three technical repeats and four biological repeats.

### HepG2 liver cell toxicity

HepG2 cells were cultured as previously described (Rajamuthiah et al., 2015). Cells were grown for either 8 or 24 h after which cell viability was assessed using the MTT [3-(4,5-dimethylthiazol-2-yl)-2,5-diphenyltetrazolium bromide] assay. Absorbance (OD_595nm_) was recorded and % toxicity was measured relative to that of the no-treatment control. Experiments were performed using two technical repeats with three biological replicates.

### Multi-passage resistance studies and whole genome sequencing

Multi-passage resistance selection studies using compound AHA-1394 were performed with one MRSA (strain LAC) and one MSSA (strain SH1000) isolate. Serial passage was performed in 15 ml tubes containing 3 ml of MHB with or without varying concentrations of AHA-1394. The tubes were incubated at 37°C for 24 h prior to each serial passage; passages were performed at 24 h intervals by transferring a 30 μl aliquot of culture containing approximately 10^6^ CFU from the tube nearest the MIC which had the same turbidity as antibiotic-free controls. When an MIC for a strain stabilized at 128 μg/ml during four successive passages, serial transfer in the presence of antibiotic was discontinued, and the strains were grown to 10 passages in antibiotic-free medium.

Genome sequencing and bioinformatic analysis was provided by MicrobesNG^1^. We chose 4 strains to sequence: Two strains that were resistant to AHA-1394 at 128 μg/ml (LAC-128 and SH1000-128) and two control strains (LAC-B and SH1000-B) that were serially passaged in parallel in the absence of AHA-1394. Genomic DNA was isolated according to MicrobesNG protocol and genome sequencing was performed on an Illumina sequencing platform with 2 × 250 bp paired end reads with 30X coverage. Accession numbers can be found here https://www.ncbi.nlm.nih.gov/bioproject/PRJNA859022.

### Relative Darwinian fitness

Competitions were established in TSB using 10^4^ CFU/mL of either SH1000-B or LAC-B and corresponding mutant strains. The bacteria were competed at 37°C with shaking (180 rpm) for 24 h. Final cell numbers were enumerated by serial dilutions on TSA plates (total cell count) and TSA plates containing 16 μg/ml AHA-1394 as an indicator for mutant cell count. The fitness of a strain was defined as a measure of the reproductive success of the population, which can be expressed as the natural logarithm of the ratio of the final and initial cell densities of the culture (Lenski et al., 1991), using the following formula:

$$\text{relative}\,\left(\text{Darwinian}\right)\,\mathrm{fitness}=\frac{\ln\,\left(A\,\left(1\right)/A\,\left(0\right)\right)}{\ln\,\left(M\,\left(1\right)/M\,\left(0\right)\right)}$$

Where *A*(0), estimated density of test strain at time 0; *M*(0), estimated density of marker strain at time 0; *A*(1), estimated density of test strain at time 1 d; *M*(1), estimated density of marker strain at time 1 d; ln, natural logarithm (logarithm to the base *e*).

### FITC-poly-L-lysine and cytochrome C binding assays

A FITC-poly-L-lysine (FITC-PLL) binding assay was used to determine the relative surface charge of the bacteria. Overnight *S. aureus* cultures where diluted to an OD_600nm_ 0.5 in 1 ml of PBS. These suspensions were subsequently incubated with 80 μg/ml FITC-PLL (MW 15,000–30,000; Sigma) for 10 min at room temperature in the dark. Cells were then washed three times in 1 ml of PBS by three rounds of centrifugation (16,000 × *g* for 1 min). Fluorescence was visualized by using a PHERAstar (BMG Labtech) plate reader (excitation 485 nm: emission 525 nm). The FITC-poly-L-lysine binding assay was compared with another commonly used method to investigate differences in surface charge, a cytochrome C binding assay. For the cytochrome C binding assay, overnight cultures were normalized to an OD_600nm_ of 8. The bacterial suspensions were washed twice in MOPS buffer (20 mM pH 7.0) and finally resuspended in 200 μl of MOPS buffer. Samples were then combined with 50 μl of cytochrome C (equine heart, 2.5 mg/ml in MOPS buffer) and incubated for 10 min at room temperature. Finally, samples were pelleted (16,000 × *g* for 1 min) and 200 μl of supernatant read for absorbance at Abs_530nm_ using a SUNRISE Tecan microplate reader.

### Transmission electron microscopy

*S. aureus* strains SH1000-B, SH1000-128, SH1000-128 *mprF::Tn*, LAC-B and LAC-128 were imaged by transmission electron microscopy (TEM) to compare differences in cell wall thickness, using a previously described method (Grigor’eva et al., 2020). Briefly, 1 ml of 18 h overnight cultures grown in TSB were pelleted and resuspended in 500 μl of 4% paraformaldehyde, 2.5% glutaraldehyde mixture. Samples were subsequently incubated at room temperature for 20 min before being pelleted and stored for 24 h at 4°C. Following this, samples were washed to remove fixative, postfixed in a 1% osmium tetroxide solution for 1 h and dehydrated in ethanol acetone. Samples were then embedded in an epon-araldite mixture and left to polymerize at 60°C for 24–48 h to obtain hard blocks. Blocks were sectioned (70 nm) using a Leica UC7 ultramicrotome and imaged using a FEI Tecnai T12 microscope at the Wolfson Bioimaging Facility, University of Bristol. For cell wall thickness analysis, 50 cells were measured for each strain, with each individual cell being measured in 4 equidistant locations before being averaged. Similarly, 50 cells were measured for each strain for the cell area analysis. The diameter was measured across 2 perpendicular planes before being averaged, the radius calculated, yielding cell area values by *A* = π *r*^2^.

### Synergistic activity with clinically relevant antibiotics

The ability of AHA-1394 to synergize with daptomycin, oxacillin, vancomycin and linezolid was tested using the microbroth dilution method. Overnight *S. aureus* cultures were diluted 1:200 in MHB and grown to early exponential phase (OD_600nm_) of 0.5–0.6. The final concentration was 256 μg/ml for oxacillin, 8 μg/ml for daptomycin, 8 μg/ml for linezolid and either 8 μg/ml or 16 μg/ml for vancomycin depending on the strain tested. Following 18 h incubation at 37°C the sum of the fractional inhibitory concentration (ΣFIC) was calculated for each well that corresponded to a minimal inhibitory concentration (MIC) using the equation: ΣFIC = FIC_*A*_ + FIC_*B*_ = (MIC_A+B combination_/MIC_*A*_) + (MIC_B+A combination_/MIC_*B*_), where MIC_A_ and MIC_*B*_ are the MICs of drugs A and B alone. The FIC index value presented represents the lowest value achieved for each polyamine/drug combination. The interpretation of the FIC index was designated as either synergistic (S, FIC ≤ 0.5), additive (A, FIC >0.5 < 1), or indifferent (I, FIC ≥ 1 < 4).

### Statistical analysis

Paired two-tailed Student’s *t*-test or one-way analysis of variance (ANOVA) with a Dunnett’s multiple comparisons test, with single pooled variance (GraphPad Prism v9.0), were used to analyze the observed differences between experimental results. A *P*-value < 0.05 was considered statistically significant.

## Results

### AHA-1394 displays antimicrobial activity against an extended panel of MSSA, MRSA, VISA and VRSA *S. aureus* strains

In this study we synthesized three novel linear synthetic polyamines designated AHA-1282, AHA-1268 and AHA-1394 with an increased total net charge compared to their monomeric counterparts. To test the activity of these new compounds, the MIC was compared to spermine, nor-spermine, spermidine and nor-spermidine against SH1000, an MSSA laboratory strain (Table 2 and Supplementary Figure 1). SH1000 was able to grow in the presence of all four of the naturally occurring polyamines up to concentrations of 256 μg/ml. Both AHA-1282 and AHA-1268 displayed an MIC of 32 μg/ml, a greater than eightfold increase in activity whereas AHA-1394 displayed the most potent anti-staphylococcal activity with an MIC of 2 μg/ml, a greater than 64-fold increase in activity. MIC of AHA-1394 was further tested using a cohort of clinically relevant, multi-drug resistant and genetically diverse strains summarized in Table 1, with MIC values ranging from 2 to 8 μg/ml (Table 2 and Supplementary Figure 2). The minimum bactericidal concentration of AHA1394 against both MSSA (SH1000) and MRSA (LAC) was determined as 16 μg/ml (Supplementary Figure 3).

**TABLE 2:** Novel linear polyamines display enhanced bactericidal activity against *S. aureus*.

MIC (g/ml)	SH1000	AHA-1394 (MIC (g/ml))	*S. aureus* strain
Spermine	> 256	8	Newman
Norspermine	> 256	2	MRSA252
Spermidine	> 256	2	TW20
Nor-spermidine	> 256	2	EMRSA15
AHA-1282	32	4	LAC
AHA-1268	32	4	MW2
AHA-1394	4	2	Mu3
		2	Mu50

MICs were performed in biological triplicate for spermine, nor-spermine, spermidine, nor-spermidine, and synthetic derivatives AHA-1282, AHA-1268 and AHA-1394 against SH1000.

MICs were also performed in biological triplicate for AHA-1394 against a panel of clinical isolates with differing antibiotic resistance profiles MICs were defined as the lowest concentration resulting in complete inhibition of growth.

### AHA-1394 is a potent biofilm inhibitor

**FIGURE 2:**
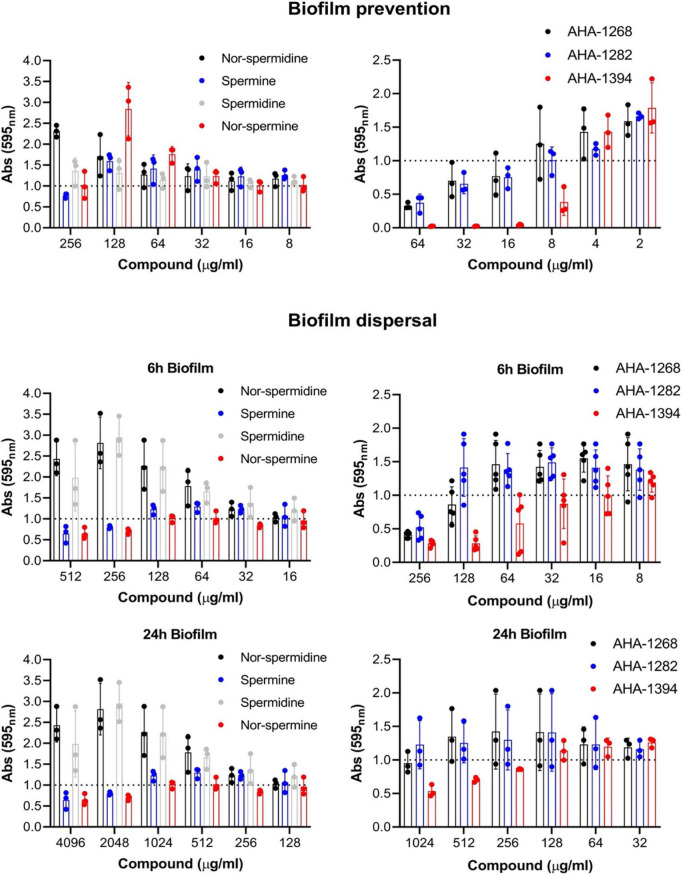
AHA-1394 performs better than natural polyamines in biofilm inhibition. The biofilm prevention and dispersal activity of natural polyamines were compared to AHA-1268, AHA-1282 and AHA-1394. For biofilm prevention polyamines were added at a final concentration ranging 256–2 μg/ml, whereas for biofilm dispersal polyamines were added at a final concentration ranging 4,096–8 μg/ml. Absorbance 595_*nm*_ values of the polyamines below the normalized threshold of 1.0 showed biofilm preventative and dispersal activity. The dots represent biological replicates with the bars representing the mean and error bars the standard deviation.

We investigated whether the synthetic polyamines displayed enhanced biofilm inhibitory activity against the strong biofilm-forming isolate, SH1000 (Figure 2 and Supplementary Figure 4). Of the natural polyamines, only spermine displayed limited biofilm reduction (20%) at 256 μg/ml. Comparably, AHA-1268 and AHA-1282 reduced biofilm formation by greater than 50% at 64 μg/ml, whereas AHA-1394 displayed complete biofilm inhibition at a concentration of 16 μg/ml. We were also interested in whether these compounds could penetrate and contribute to biofilm dispersal. SH1000 biofilm was allowed to form for 6 h or 24 h followed by the addition of the relevant polyamines and a further incubation for 24 h to allow for biofilm dispersal (Figure 2). Spermine and nor-spermine displayed limited biofilm reduction (20%) for both 6 h and 24 h biofilms at 256 μg/ml and 2,048 μg/ml respectively. AHA-1268 and AHA-1282 caused a 50% reduction in 6 h biofilm formation at 256 μg/ml with AHA-1394 displaying 50% biofilm reduction at 128 μg/ml. AHA-1394 also performed best at biofilm dispersal of 24 h biofilms, where a 50% reduction was observed following treatment with 1,024 μg/ml.

### AHA-1394 displays minimal toxicity against human cells

**FIGURE 3:**
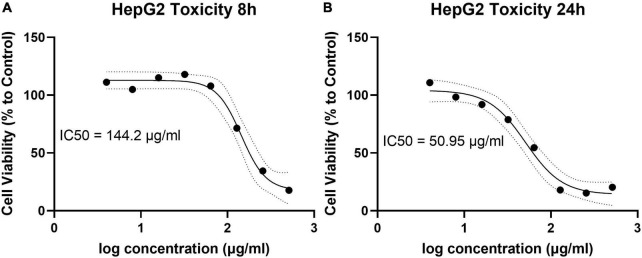
AHA-1394 HepG2 cytotoxicity and IC_50_. Log AHA-1394 concentration was plotted against the percentage of live HepG2 cells at **(A)** 8 h and **(B)** 24 h following normalization of HepG2 cells incubated without AHA-1394. The IC_50_ was calculated according to standard curve interpretation and defined as the concentration responsible for 50% HepG2 cell death.

The cytotoxicity of the most active synthetic polyamines, AHA-1394, toward the human hepatocellular carcinoma cell line (HepG2) was evaluated at both 8 h and 24 h incubation (Figure 3). The IC_50_ of AHA-1394 against HepG2 cells was 144.2 μg/ml and 50.95 μg/ml following 8 h and 24 h incubation respectively. The MIC_50_ (defined as the median concentration for which 50% of the isolates were inhibited (Schwarz et al., 2010) for AHA-1394 from the 10 *S. aureus* strains tested was 2 μg/ml giving a selectivity index (defined as IC_50_/ MIC_50_ (Ong et al., 2013)) of 25.48, providing a robust therapeutic window, indicating that AHA-1394 is selective for bacteria over human cells.

### AHA-1394 is active against previously described polyamine resistant strains

*S. aureus* polyamine resistance involves either the acquisition of the *speG* N-1 acetyltransferase or spontaneous mutation resulting in small colony variant formation (SCV) (Joshi et al., 2011). To test whether these two polyamine resistance mechanisms could be associated with a loss of susceptibility to AHA-1394, MICs were performed against JE2 WT and isogenic *speG* transposon mutant, and Newman WT and *menD* SCV associated mutant. Inactivation of *speG* had no effect on the susceptibility toward AHA-1394 with both JE2 and JE2 *speG::Tn* having an MIC of 8 μg/ml, suggesting the inability of SpeG to acetylate this novel polyamine. Deletion of *menD* in the Newman background was associated with a slight increase of MIC from 8 μg/ml to 16 μg/ml.

### Fitness cost associated with AHA1394 resistance

**FIGURE 4:**
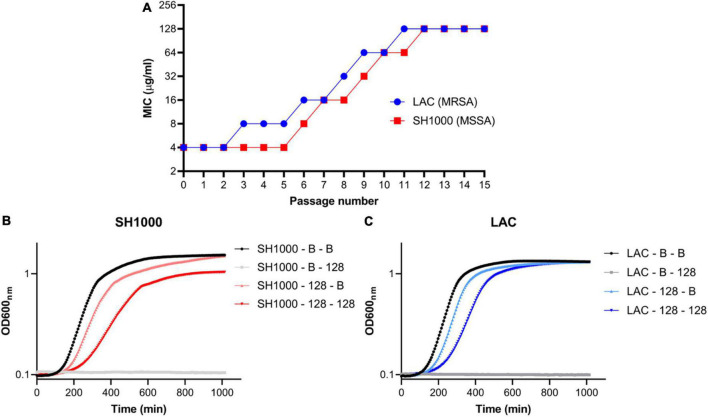
Stable AHA-1394 resistant mutants emerge following AHA-1394 serial passage. **(A)** Increase of MIC following passage every 24 h of LAC (blue) and SH1000 (red). Bacterial growth curves of **(B)** SH1000-B and SH1000-128 and **(C)** LAC-B and LAC-128 grown in MHB (SH1000-B-B; SH1000-128-B; LAC-B-B; LAC-128-B) or MHB containing AHA-1394 (128 μg/ml) (SH1000-B-128; SH1000-128-128; LAC-B-128; LAC-128-128). OD600_*nm*_ was plotted against time following 18 h incubation. The dots represent the mean of three biological replicates.

Two *S. aureus* strains (LAC and SH1000) were serially passaged in increasing concentrations of AHA-1394 up to 128 μg/ml, in parallel to broth controls (Figure 4A). This resulted in the generation of two stable, resistant strains denoted LAC-128 and SH1000-128 after 11 and 12 passages respectively. Spontaneously acquired resistance mechanisms are often associated with characteristic growth defects and fitness costs, typified by SCVs (Proctor et al., 2006). Therefore, the relative growth kinetics of LAC-128 and SH1000-128 were compared to their serially passaged broth controls, denoted LAC-B and SH1000-B (Figures 4B,C). LAC-128 and SH1000-128 displayed increased generation times (doubling time = ln2/*k*) of 3.4 min and 13.2 min respectively, compared to control strains when grown in TSB, confirming an inherent growth defect, suggesting AHA-1394 resistance is energetically costly. Accordingly, the relative Darwinian fitness of LAC-128 and SH1000-128 were calculated following 18 h growth. Both SH1000-128 and LAC-128 showed a marked decrease in relative fitness, with mean values of 0.692 (SD = 0.008) and 0.696 (SD = 0.035) respectively.

### Resistance to AHA-1394 is associated with gain of function mutations in *mprF*

**FIGURE 5:**
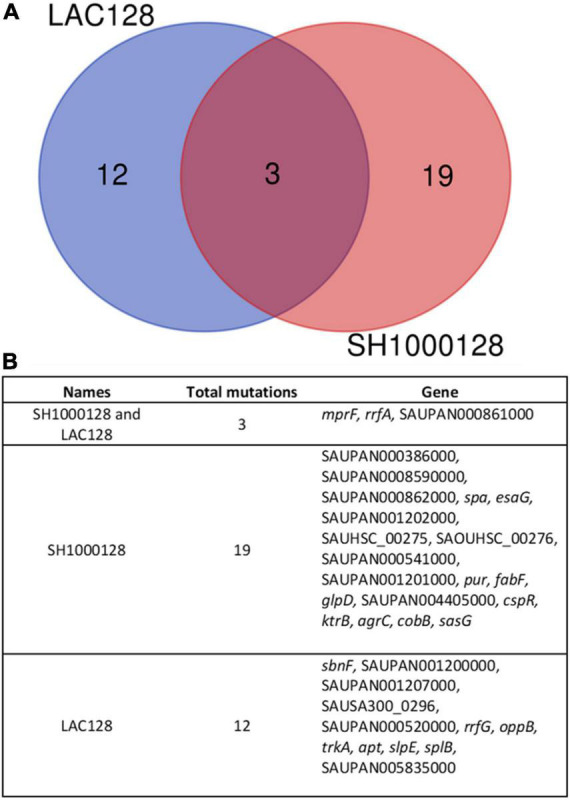
Comparison of mutations indicate a role for MprF in AHA-1394 resistance. **(A)** Venn diagram showing mutations unique to LAC-128 (blue), unique to SH1000-128 (red) and mutation shared by both mutants (mauve) when compared to the parental strains LAC-B and SH1000-B. **(B)** Descriptions of mutations present and shared in LAC-128 and SH1000-128. Pangenome locus tags are shown (“SAUPAN”). Where a pangenome locus tag is missing the relevant USA300 “SAUSA300” or NCTC8325 “SAOUHSC” locus tag is used.

To investigate the mechanism of AHA-1394 resistance, LAC-128 and SH1000-128, alongside respective controls (LAC-B and SH1000-B) were genome sequenced. Subsequent pairwise single nucleotide polymorphism (SNP) comparison between resistant and sensitive controls identified non-synonymous mutations within resistant mutants (22 for SH1000-128 and 15 for LAC-128) (Figure 5A). Both LAC-128 and SH1000-128 had mutations in three genes: *mprF, rrfA* and SAUPAN00861000 (Figure 5B). The exact *rrfA* and SAUPAN00861000 mutation differed between LAC-128 and SH1000-128, however, both contained the same S337L *mprF* mutation, indicating a gain of function *mprF* mutation may be responsible for AHA-1394 resistance. To confirm, we performed AHA-1394 MIC experiments using wild-type JE2 and isogenic JE2 *mprF*::*Tn*. JE2 displayed an MIC of 8 μg/ml, whereas JE2 *mprF::Tn* was more susceptible to AHA-1394 with an MIC of 4 μg/ml. We next phage transduced the *bursa aurealis* transposon using Φ11 from JE2 *mprF::Tn* into two recipient strains: SH1000-B and SH1000-128. AHA-1394 MICs were repeated on SH1000-B, SH1000-B *mprF::Tn*, SH1000-128 and SH1000-128 *mprF::Tn.* Both SH1000-B *mprF::Tn* and SH1000-128 *mprF::Tn* were more susceptible to AHA-1394 killing with MICs of 2 μg/ml and 8 μg/ml compared with 4 μg/ml and > 128 μg/ml for SH1000-B and SH1000-128 respectively. Importantly, deletion of *mprF* restored the susceptibility of SH1000-128 to one doubling above SH1000-B, indicating the importance of *mprF* mutation S337L in AHA-1394 resistance.

### *mprF* S337L mutation is associated with altered surface charge in strain LAC

**FIGURE 6:**
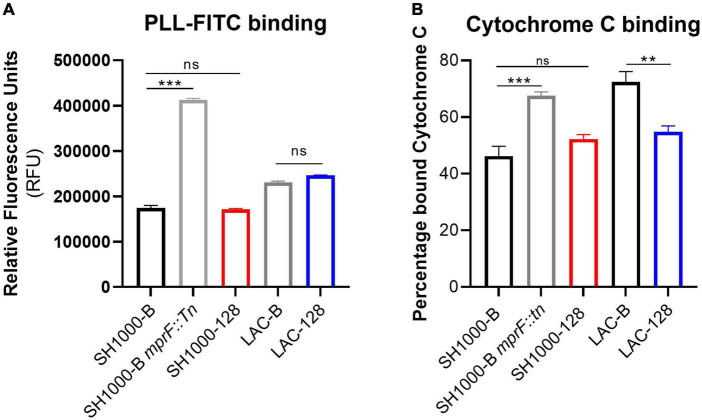
S337L is associated with altered cell surface charge in strain LAC. An indication of cell surface charge was estimated through the binding ability of **(A)** PLL-FITC, shown by relative fluorescence units (RFU) normalized to a PBS blank and **(B)** cytochrome C binding depicted as percentage of bound cytochrome C normalized to a MOPS blank. The bars represent the mean of three biological replicates and the error bars the standard deviation. A one-way ANOVA was performed on SH1000-B, SH1000-B *mprF::Tn*, and SH1000-128, whereas a paired Student’s *t*-test was performed between LAC-B and LAC-128. “ns” denotes not significant, significance was determined as the following *p* values: **0.01; ***0.001.

Mutations in *mprF* that confer elevated daptomycin MIC are classically associated with increased surface charge due to elevated levels of lysyl-phosphatidylglycerol in the bacterial membrane (Jones et al., 2008). To examine whether the S337L mutation observed in AHA1394 resistant strains resulted in an increase in membrane charge, both an FITC-poly-l-lysine and cytochrome C binding assay were employed. In agreement with the literature, a significant increase in FITC-poly-l-lysine and cytochrome C binding was detected for the SH1000-B *mprF::Tn* control strain confirming the suitability of both assays (Figure 6) (Ernst et al., 2018). Interestingly, there was no notable difference in the FITC-poly-l-lysine binding ability of either LAC-128 or SH1000-128 compared to LAC-B and SH1000-B (Figure 6A), whereas a significant difference in bound cytochrome C was observed only between the LAC-B and LAC-128 and not in the SH1000 background. This suggests the S337L mutation present in LAC-128 may alter the levels of lysyl-phosphatidylglycerol in the membrane. We also sought to test whether the S337L mutation contributed to daptomycin non-susceptibility (DNS) in these two backgrounds by performing daptomycin MICs. The growth of SH1000-B and LAC-B were inhibited at 1 μg/ml, while SH1000-128 and LAC-128 could survive at higher daptomycin concentrations of > 8 μg/ml and 4 μg/ml, respectively. Deletion of *mprF* in the SH1000-B background reduced the MIC to 0.25 μg/ml while deletion of *mprF* in the SH1000-128 also increased daptomycin susceptibility to 2 μg/ml confirming the involvement of S337L in conferring daptomycin resistance.

### *mprF* S337L mutation is associated with increased cell wall thickness

**FIGURE 7:**
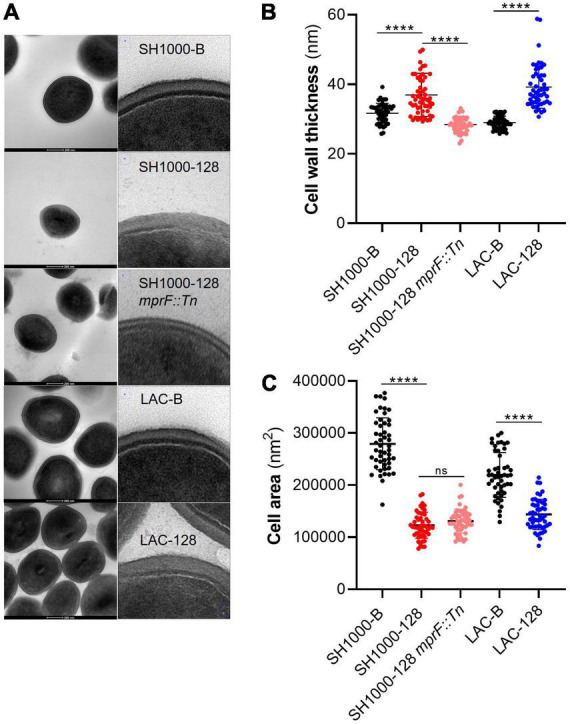
AHA-1394 resistance is associated with increased cell wall thickness. **(A)** TEM images of each strain at 300,000x magnification are shown with a 200 nm scale bar. For each strain zoomed in images of just the cell wall are shown on the right. **(B)** The cell wall thickness in nm of 50 individual cells for each of the strains SH1000-B, SH1000-128, SH1000-128 *mprF::Tn*, LAC-B and LAC-128. The horizontal line represents the mean and the error bars the standard deviation. **(C)** The cell area in nm^2^ of 50 individual cells for the same panel of strains. The horizontal line represents the mean and the error bars the standard deviation. A one-way ANOVA was performed on SH1000-B, SH1000-128, SH1000-128 *mprF::Tn*, whereas a paired Student’s *t*-test was performed between LAC-B and LAC-128. “ns” denotes not significant, significance was determined as the following *p* values: ****0.0001.

As SH1000-128 did not display reduced FITC-poly-l-lysine or cytochrome C binding we wanted to investigate other factors that may underline AHA-1394 resistance. Previous work has indicated that certain *mprF* mutations are linked to the over expression of the two-component regulatory system, *vraRS*, conferring an increase in cell wall thickness resulting in decreased susceptibility to daptomycin (Mehta et al., 2012). Accordingly, we investigated cell wall thickness and cell area in the sensitive, resistant and SH1000-128 *mprF::Tn* mutant strains using transmission electron microscopy (TEM) (Figure 7A). We observed that both SH1000-128 and LAC-128 had significantly thicker cell walls with mean cell wall thickness values of 36.94 nm (SD = 6.23 nm) and 39.24 nm (SD = 6.12 nm), respectively, compared to SH1000-B and LAC-B which displayed cell wall thickness values of 31.67 nm (SD = 2.84 nm) and 28.98 nm (SD = 2.82 nm), respectively (Figure 7B). Interestingly, we found that the increase in cell wall thickness exhibited by the SH1000-128 polyamine resistant strain was ablated when *mprF* was deleted. This provides insight into the potential role of *mprF* in regulating cell wall homeostasis.

When performing the cell wall thickness analysis, we noticed that both SH1000-128 and LAC-128 appeared to be smaller. Subsequent analysis of cell area (Figure 7C) indeed showed that both resistant strains were significantly smaller. Importantly, although deletion of *mprF* in SH1000-128 restored cell wall thickness to near WT-levels, the same effect was not seen for cell area, perhaps indicating that other mutations could be causing this phenotype. Importantly, increased cell wall thickness and altered cell area seems to reaffirm why we observed a decrease in growth rate and relative Darwinian fitness in the AHA-1394 resistant strains.

### AHA-1394 synergizes with clinically relevant anti-staphylococcal antibiotics

Previous studies have indicated that natural polyamines synergize with and increase the susceptibility to certain antibiotics (Kwon and Lu, 2007; Liu et al., 2018). Accordingly, synergy measurement by checkerboard MIC analysis were performed using AHA-1394 partnered with the clinically important anti-staphylococcal antibiotics; oxacillin, daptomycin, vancomycin, and linezolid against a selection of clinical isolates (Table 3 and Supplementary Figure 4). We show an impressive ability of AHA-1394 to synergize with oxacillin against all six strains tested as indicated by FIC index. The oxacillin MIC was reduced from > 256 μg/ml to 16 μg/ml (LAC), 16 μg/ml (EMRSA15), 4 μg/ml (MRSA252), 16 μg/ml (TW20), 8 μg/ml (Mu3), and 8 μg/ml (Mu50) with the addition of 0.5–1 μg/ml of AHA-1394. Furthermore, AHA-1394 was able to potentiate the activity of vancomycin against strains TW20, Mu3 and Mu50, restoring vancomycin susceptibility for the VISA strains Mu3 and Mu50 from an initial MIC of 4 μg/ml and 8 μg/ml to 1 μg/ml and 0.5 μg/ml with the addition of 0.5–1 μg/ml of AHA-1394. Strain dependent synergy was also observed for daptomycin partnered with AHA-1394, restoring daptomycin susceptibility toward TW20, Mu50 and SH1000, decreasing the MIC from 4 μg/ml, 8 μg/ml and 8 μg/ml to 0.5 μg/ml,1 μg/ml and 2 μg/ml, respectively, with the addition of 1 μg/ml of AHA-1394. Finally, AHA-1394 was tested for its synergy with the oxazolidinone linezolid, however, it did not improve the bactericidal activity of this antibiotic.

**TABLE 3:** The synergy of AHA-1394 with clinically relevant antibiotics is strain dependent.

		Combinations			
		AHA1394			
*S. aureus* strain		Oxacillin	Daptomycin	Vancomycin	Linezolid
LAC	FIC rep 1	0.28125	0.75	0.625	1
	FIC rep 2	0.28125	0.75	0.625	1
	Interpretation	S	A	A	I
EMRSA15	FIC rep 1	0.15625	0.75	1	1
	FIC rep 2	0.28125	0.5	1	1
	Interpretation	S	A	I	I
MRSA252	FIC rep 1	0.26	0.5625	0.5625	0.625
	FIC rep 2	0.28125	0.625	0.5625	0.625
	Interpretation	S	A	A	A
TW20	FIC rep 1	0.3125	0.375	0.375	0.75
	FIC rep 2	0.28125	0.375	0.375	0.75
	Interpretation	S	S	S	A
Mu50	FIC rep 1	0.2539	0.375	0.3125	0.75
	FIC rep 2	0.1328	0.375	0.25	0.75
	Interpretation	S	S	S	A
Mu3	FIC rep 1	0.2656	0.56	0.5	0.53
	FIC rep 2	0.3125	0.75	0.375	0.53
	Interpretation	S	A	S	A
SH1000	FIC rep 1	N/A	0.3125	N/A	1
	FIC rep 2	N/A	0.5	N/A	1
	Interpretation	N/A	S	N/A	I

AHA1394 was tested in combination with either oxacillin, daptomycin, vancomycin and linezolid against a selection of *S. aureus* isolates.

SH1000 was not tested for vancomycin or oxacillin synergy as it exhibited extremely low MICs against these compounds.

The interpretation of the FIC index was designated as either synergistic (S, FIC ≤ 0.5), additive (A, FIC > 0.5 < 1), or indifferent (I, FIC ≥ 1 < 4).

We next tested the activity of antibiotic combinations against the AHA-1394 resistant mutants LAC-128 and SH1000-128 using AHA-1394/oxacillin or AHA-1394/daptomycin against LAC-128 or SH1000-128. AHA-1394 showed impressive synergy with oxacillin against LAC-128 with an FIC of 0.19-0.25. However, AHA-1394 could not improve the killing activity of daptomycin against SH1000-128. Interestingly, we found that the S337L *mprF* mutation was associated with a “see-saw” effect against oxacillin, whereby increased AHA-1394 MICs were associated with a concomitant decrease in oxacillin MIC (Jiang et al., 2022). For LAC-128 and SH1000-128 the oxacillin MIC was 32 μg/ml and 0.125 μg/ml compared with 256 μg/ml and 2 μg/ml for LAC-B and SH1000-B respectively.

## Discussion

We have shown that polyamines can be used as scaffolds for the design of novel antimicrobial compounds effective against *S. aureus.* We hypothesize that increasing the positive charge along the linear polyamines leads to an increase in their electrostatic attraction to the *S. aureus* lipid bilayer culminating in enhanced bactericidal activity and biofilm prevention as observed with compound AHA-1394 against a panel of multidrug resistant *S. aureus*.

*S. aureus* resistance to polyamines has been described and linked to the acquisition of the arginine catabolic mobile element (ACME), housing the N-1 spermidine acetyltransferase SpeG (Joshi et al., 2011). It is unclear how SpeG-mediated acetylation of polyamines prevents toxicity to *S. aureus*, however, it is proposed the enzyme acts intracellularly, modifying polyamines that are rapidly imported preventing their modification into toxic intermediates (Kramer et al., 2008). Acquisition of ACME distinguishes the highly virulent USA300 community-associated (CA)-MRSA lineage from other CA- MRSA lineages and has been proposed to have contributed to the hypervirulence, hyper-transmissibility, and increased propensity to cause skin and soft tissue infections (SSTIs) of USA300 (Diep et al., 2006; Thurlow et al., 2013). Importantly, AHA1394 was effective against ACME-positive strain (LAC) as well as a panel of genetically diverse, virulent, and clinically relevant isolates suggesting that SpeG is unable to acetylate AHA-1394. Polyamine resistance can also arise through mutations of the menadione biosynthesis machinery and generation of SCV. Accordingly, we tested the susceptibility of a previously constructed *menD* SCV mutant and observed an increase in MIC from 8 to 16 μg/ml. Considering SCVs are typically associated with a fourfold increase in MIC against β-lactams and a 4–8-fold increase in MIC against aminoglycosides (R. A. Proctor et al., 1995; Proctor and von Humboldt, 1998; Proctor et al., 2006), our data indicates the therapeutic potential of AHA-1394 against difficult to eradicate *S. aureus* SCVs.

An important aspect of any antibacterial candidate is the selectivity of the drug to bacteria over human cells. Accordingly, we tested the toxicity of AHA-1394 using the highly sensitive liver-derived HepG2 cell line. We determined the IC_50_ of AHA-1394 at 24 h to be 50.95 μg/ml, which is comparable to many current antibiotics approved for use in the clinic; azithromycin (Abdel-Hamid et al., 2017) and linezolid (Sun et al., 2019) commonly used to treat *S. aureus* SSTIs have HepG2 IC_50_s of 42.8 μg/ml, 10.1 μg/ml, respectively.

Resistant mutants were selected using a broth multi-passage experiment. Repeated exposure for 11 (LAC) and 12 (SH1000) days resulted in a 32-fold increase in MIC. This is comparable to clinical used anti-staphylococcal antibiotics; ciprofloxacin, mupirocin and fusidic acid MICs increased 256-, 64-, and 256-fold, respectively, following 25 passages (D’Lima et al., 2012). Genome sequencing of resistant mutants LAC-128 and SH1000-128 identified a common *mprF* S337L mutation. MprF is a bifunctional enzyme; the synthase domain functions as a phosphatidylglycerol lysyltransferase, while the flippase domain mediates the transfer of newly synthesized lysyl-phosphatidylglycerol from the inner to the outer leaflet of the bacterial membrane (Ernst et al., 2009). The S337L mutation present in both LAC-128 and SH1000-128 is located within the overlapping region of the synthase and flippase domains (Jiang et al., 2022). We show that S337L confers increased resistance to AHA-1394, contributes to DNS and resistance is abolished upon deletion of *mprF::*S337L.

Gain of function mutations in MprF are suspected to cause either increased lysyl-phosphatidylglycerol synthase or flippase activity which contributes to greater levels of lysyl-phosphatidylglycerol in the outer leaflet of the membrane, serving to repel positively charge antimicrobials (Ernst and Peschel, 2011; Bayer et al., 2013). As both daptomycin and AHA-1394 are cationic we reasoned that increased positive charge as a result of S337L mutation conferred resistance. Using a FITC-poly-l-lysine binding assay, we found no difference in surface charge for the AHA-1394 resistant mutants. However, analysis of surface charge *via* cytochrome C binding analysis revealed a significant difference in strain LAC-128 compared to LAC-B, where the resistant mutant displayed reduced cytochrome C binding and thus increased positive charge. MprF mutations that cause DNS but that are not associated with a detectable difference in lysyl-phosphatidylglycerol abundance have been reported (Mehta et al., 2012; Ernst et al., 2018; Jiang et al., 2022). Mehta et al. described isogenic daptomycin sensitive and resistant strains which displayed no difference in the levels of cytochrome c binding, despite containing MprF mutations (Mehta et al., 2012). These MprF mutations, of which S337L was included, did contribute to increased *vraRS* expression, and corresponded to elevated vancomycin MICs. Increased *vraRS* expression in daptomycin resistant strains contributed to increased cell wall thickness, preventing access of daptomycin to the membrane. Accordingly, we investigated the cell wall thickness of AHA-1394 susceptible (SH1000-B; LAC-B) and resistant (SH1000-128l LAC-128) strains using transmission electron microscopy. Here we observed that AHA-1394 resistant strains had significantly thicker cell walls which may prevent AHA-1394 entry and access to the membrane, resulting in resistance. Investigation is currently underway to decipher the molecular mechanisms underpinning the altered cell wall dynamics associated with AHA-1394 resistance.

Alongside drug discovery, optimizing the current panel of antimicrobials and identifying synergistic combinations can aid in treatment of AMR infections and limit the development of resistance. Polyamines can increase the susceptibility of *S. aureus* to kanamycin (Wang et al., 2016) and some β-lactams (Kwon and Lu, 2007). Molecular dissection revealed that spermine directly interacted with PBP2 reducing transglycosylase activity and rendering it more sensitive to oxacillin (Yao and Lu, 2012). Here we report that AHA-1394 restores sensitivity of MRSA isolates to oxacillin and VISA and VRSA strains to vancomycin. In addition, synergy with daptomycin has been observed in a limited number of genetic backgrounds. The most impressive synergistic activity was observed in combination with oxacillin, whereby oxacillin/AHA-1394 was also active against the AHA-1394 resistant mutant LAC-128. The exact mechanism(s) underlying AHA-1394 synergism with antibiotics is not fully understood and research is ongoing to examine this further. The combinatorial effect of a possible interaction of AHA-1394 with PBP2 resulting in the observed β-lactam synergy, and the association of AHA-1394 resistance with increased oxacillin susceptibility suggests that AHA-1394 partnered with a β-lactam could be a viable treatment option for *S. aureus* disease.

## Data availability statement

The datasets presented in this study can be found in online repositories. The names of the repository/repositories and accession number(s) can be found below: NCBI; SAMN29765906, SAMN29765907, SAMN29765908, SAMN29765909.

## Author contributions

IB and ML: conceptualization, resources, and project administration.. ED, AA, TW, SL, TJW, and ML: methodology, validation, formal analysis, and investigation. ED and ML: writing first draft of the manuscript. All authors contributed to the article and approved the submitted version.

## Footnotes

1
http://www.microbesng.com
