# Supplementary material for: Antibacterial activity of novel linear polyamines against Staphylococcus aureus
Source: Front Microbiol. 2022 Aug 22;13:948343. doi: 10.3389/fmicb.2022.948343 (PMC9441809; doi:10.3389/fmicb.2022.948343)
Supplement: Supplementary file 1 [file Presentation_1.pdf]

Antibacterial activity of novel linear polyamines against *Staphylococcus aureus*

Edward J.A. Douglas[1], Abdulaziz H. Alkhzem[2], Toska Wonfor[1], Shuxian Li[1], Timothy J. Woodman[2], Ian S. Blagbrough[2] and Maisem Laabei[1*]

[1] Department of Biology and Biochemistry, University of Bath, Bath BA2 7AY, United Kingdom

[2] Department of Pharmacy and Pharmacology, University of Bath, Bath BA2 7AY, United Kingdom

*** Correspondence:**
Maisem Laabei
ml418@bath.ac.uk

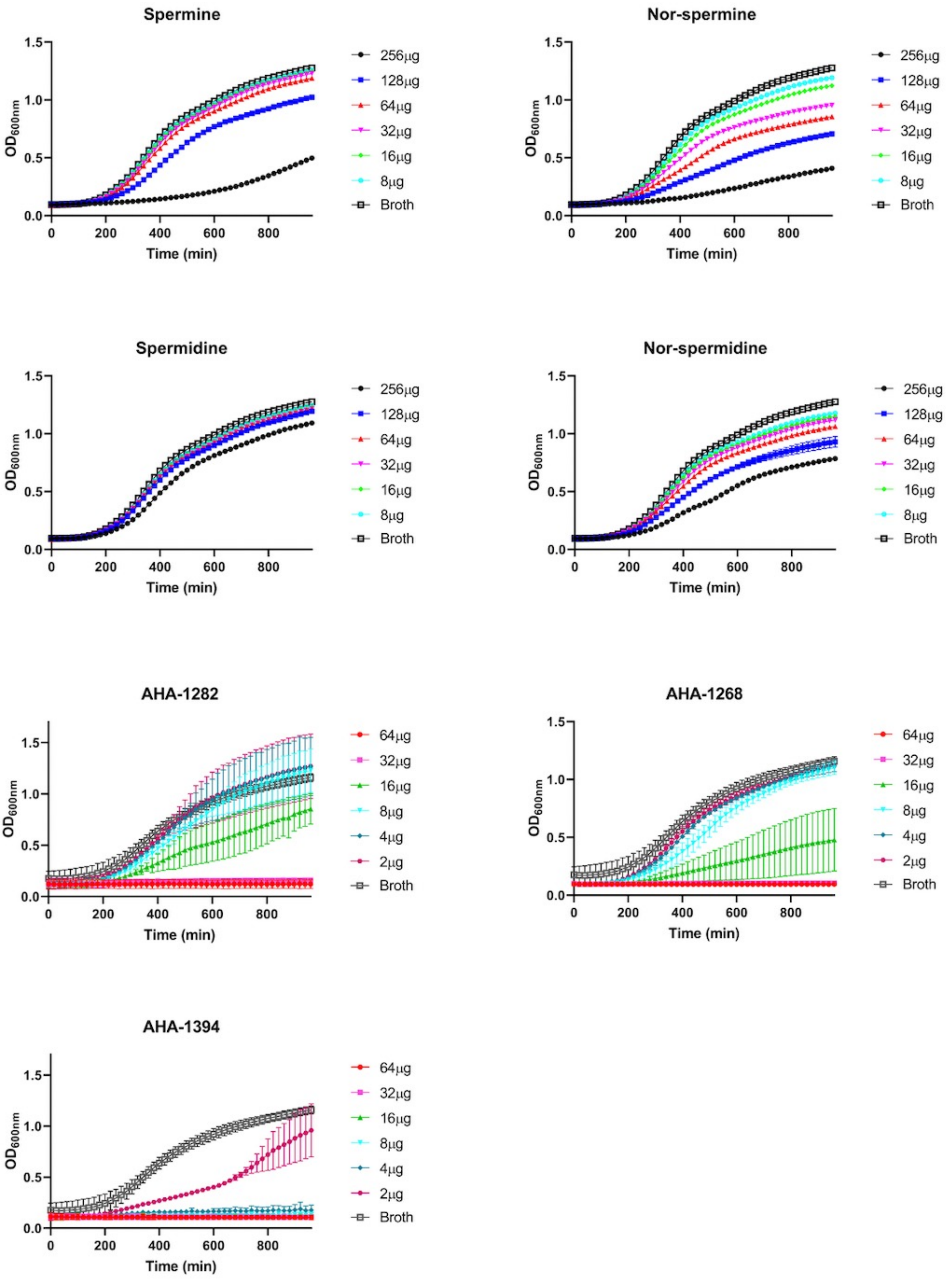

Supplementary Figure 1: MIC growth curve of natural polyamines and synthetic derivatives.
SH1000 was grown for 18 hours at 37ºC in the presence of each polyamine at a range of concentrations. The bacterial suspension was monitored for growth by measuring the OD_{600nm} every 10 min using a Tecan SUNRISE microplate reader. Growth curves were repeated in biological triplicate. The icons represent the mean and the error bars the standard deviation.

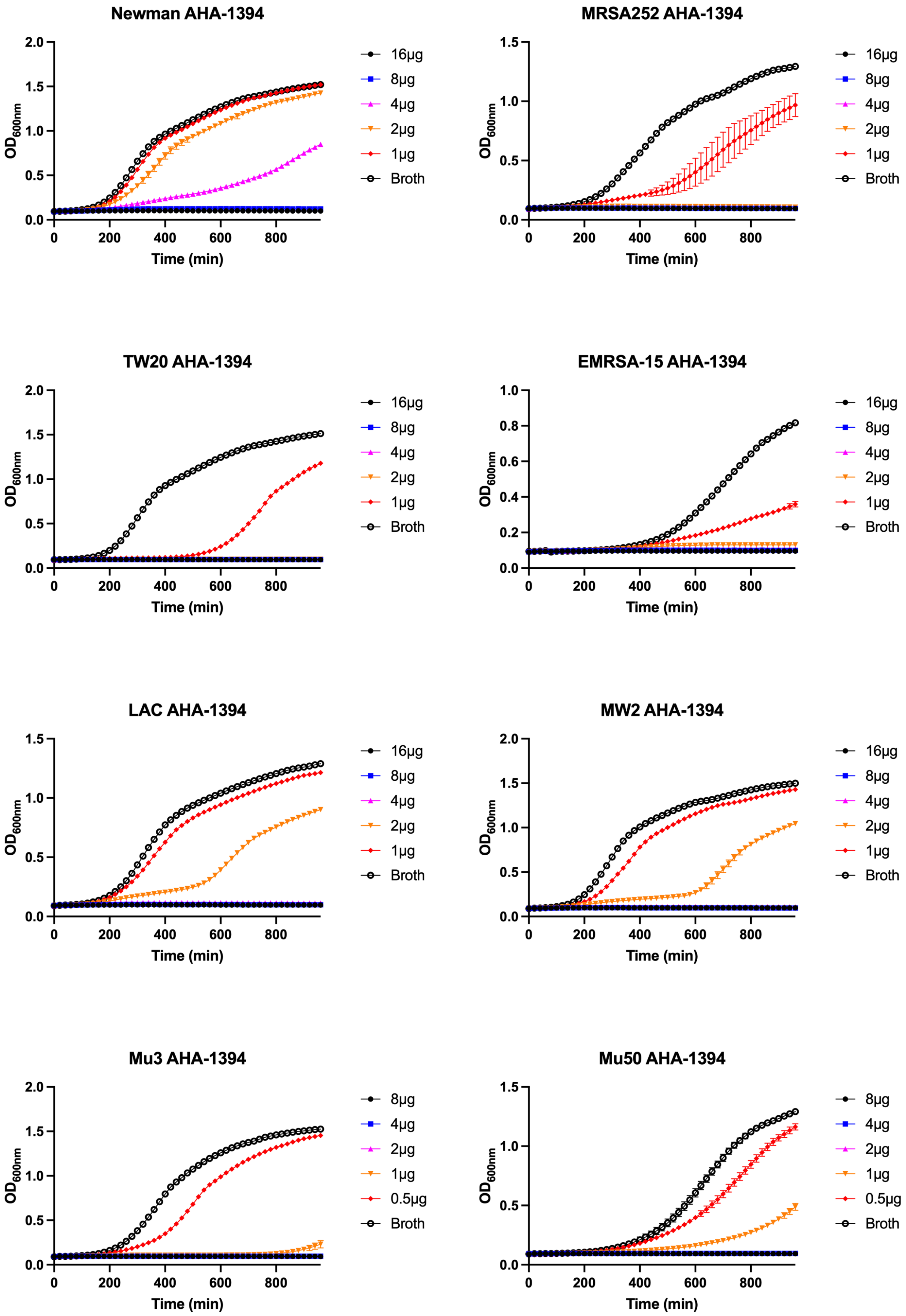

Supplementary Figure 2: MIC growth curve of AHA-1394 against a panel of clinically relevant *S. aureus* strains.

Each strain was grown for 18 hours at 37ºC in the presence of AHA-1394 at a range of concentrations. The bacterial suspension was monitored for growth by measuring the OD_{600nm} every 10 min using a Tecan SUNRISE microplate reader. Growth curves were repeated in biological triplicate. The icons represent the mean and the error bars the standard deviation.

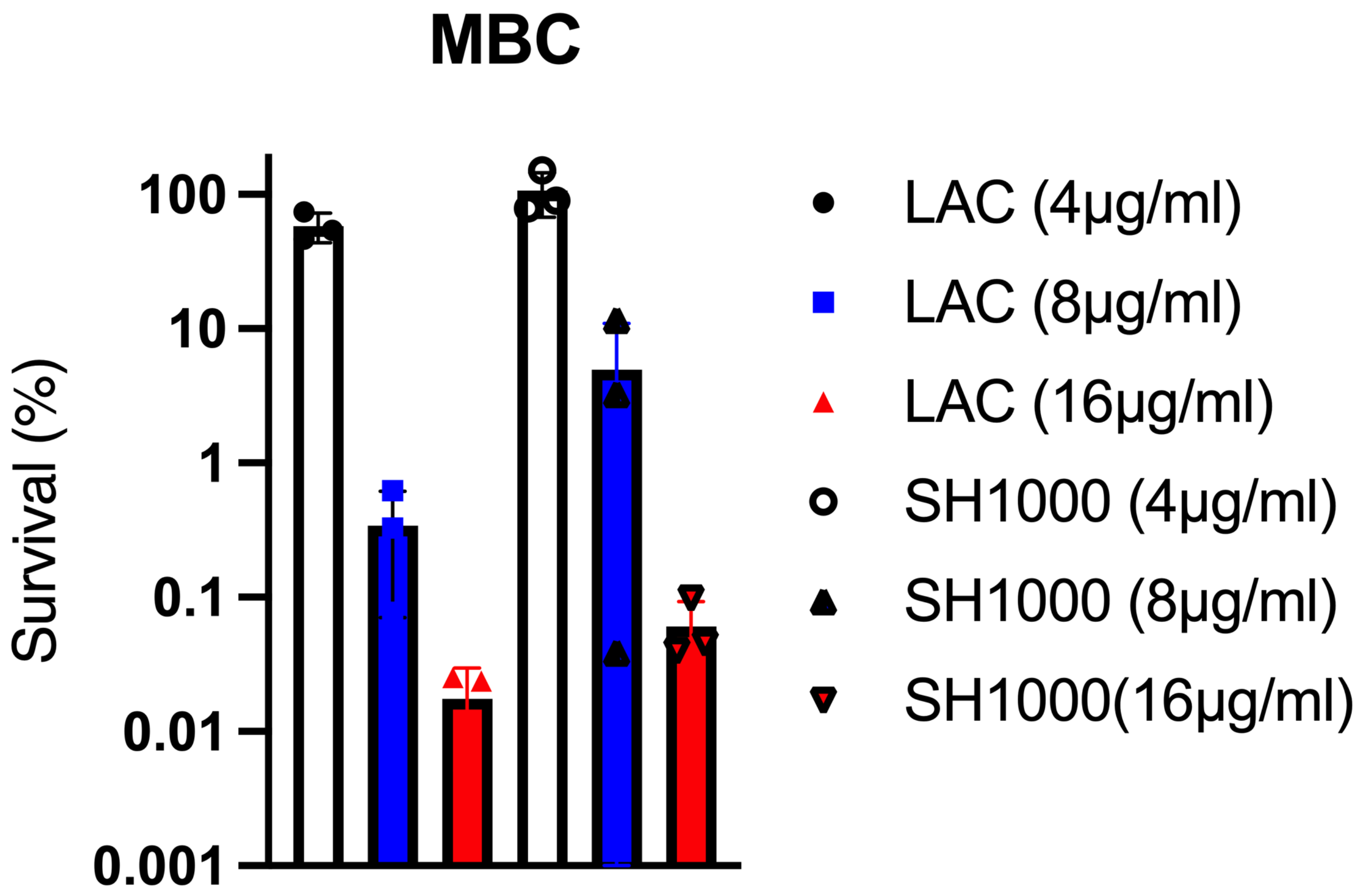

Supplementary Figure 3: Minimum bactericidal concentrations against SH1000 (MSSA) and LAC (MRSA). Following incubation of SH1000 and LAC with various concentrations of AHA-1394 as described in the MIC methodology section, ten fold serial dilution of resulting bacterial growth was performed in phosphate buffered saline and plated for CFU enumeration on TSA plates. The MBC was determined as the concentration that prevented growth and reduced the inoculum by ≥ 99.9% within 24 h. This was found to be 16 μg/ml for both LAC and SH1000. The experiment was repeated in biological triplicate as shown by individual icons. The bar represents the mean and an indication of variability is shown by the standard deviation.

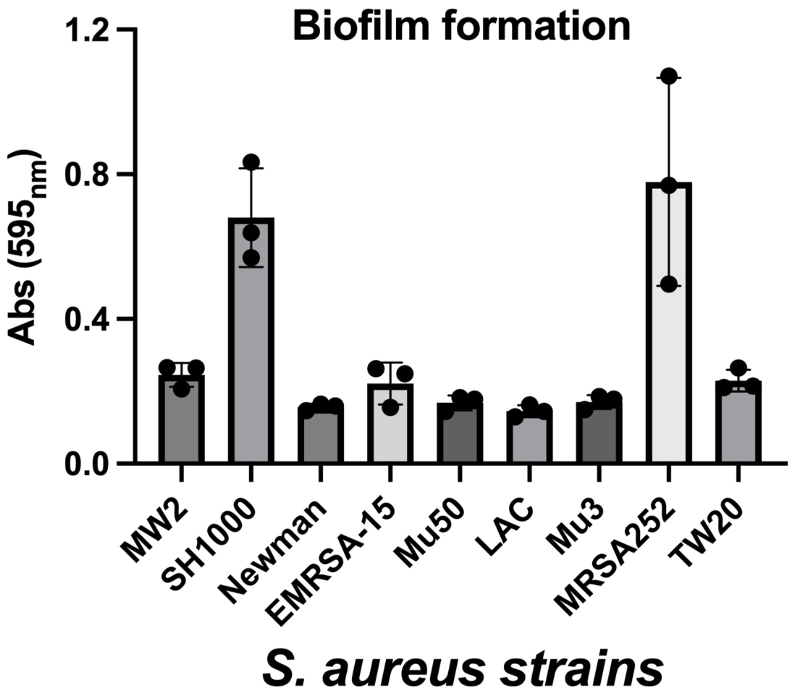

Supplementary Figure 4: Biofilm formation of selected reference MSSA and MRSA strains.
Biofilm formation of reference *S. aureus* strains was performed in TSB supplemented with 0.5% glucose as described in the methods section. Biofilm formation was determined using the crystal violet assay. Experiments were performed with three technical and three biological repeats.

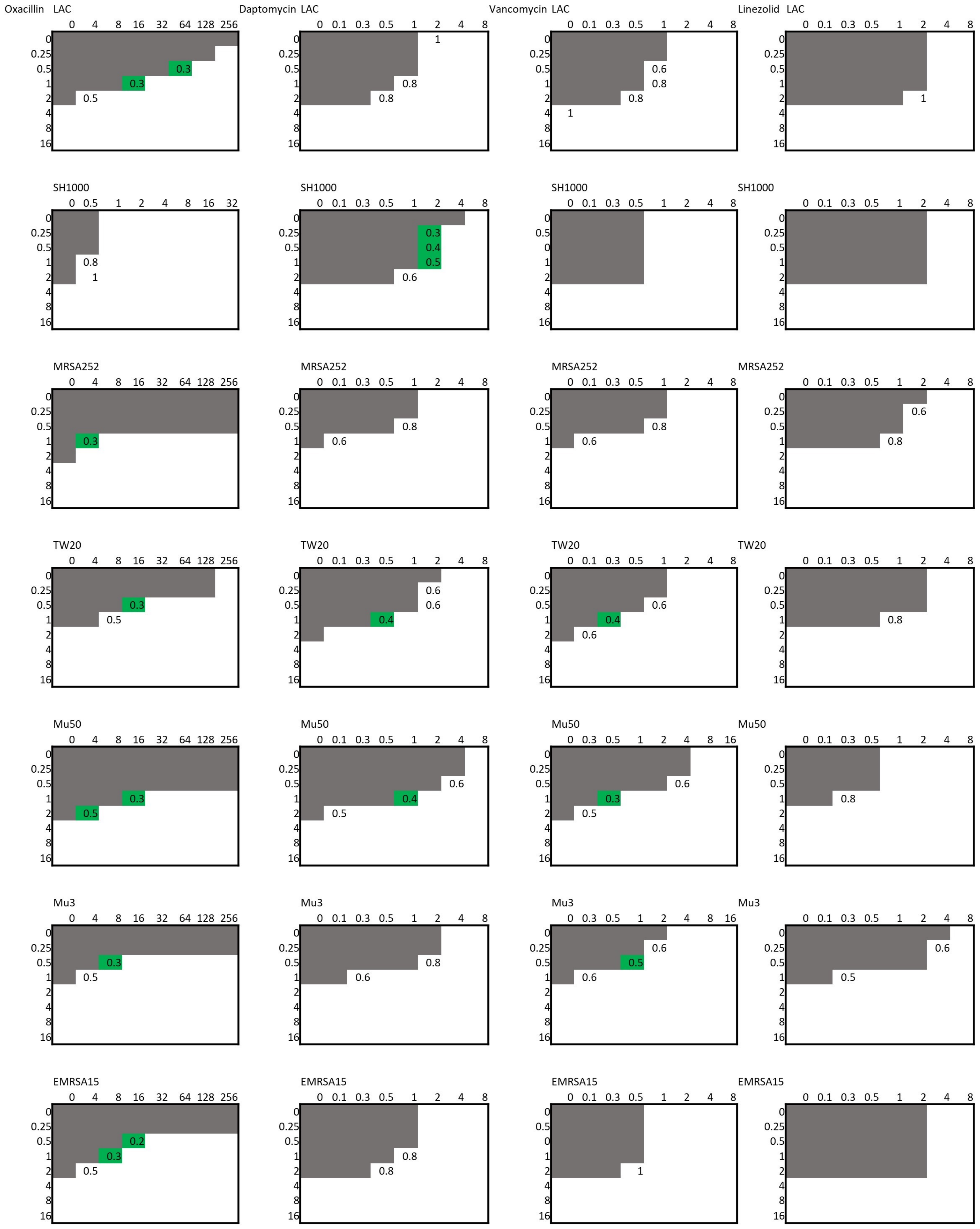

Supplementary Figure 5. Chequerboard testing of AHA-1394 partnered with clinically relevant antibiotics.

Numbers on the y-axis denote the range of concentration of AHA-13394 in μg/ml. Numbers on the x axis denotes the concentration of either oxacillin (left), daptomycin (left middle), vancomycin (right middle), and linezolid (right) in μg/ml. The presence of grey in a square denotes bacterial growth and white denotes no growth. Numbers in squares are the FIC index and squares highlighted green denote synergy as determined by an FIC of ≤0.5.
